# Novel oxazolinoanthracyclines as tumor cell growth inhibitors—Contribution of autophagy and apoptosis in solid tumor cells death

**DOI:** 10.1371/journal.pone.0201296

**Published:** 2018-07-24

**Authors:** Aneta Rogalska, Arkadiusz Gajek, Małgorzata Łukawska, Irena Oszczapowicz, Agnieszka Marczak

**Affiliations:** 1 Department of Medical Biophysics, Institute of Biophysics, Faculty of Biology and Environmental Protection, University of Lodz, Lodz, Poland; 2 Department of Modified Antibiotics, Institute of Biotechnology and Antibiotics, Warsaw, Poland; University of South Alabama Mitchell Cancer Institute, UNITED STATES

## Abstract

Chemical modification of known, effective drugs are one method to improve the chemotherapy of tumors. We reported ability of oxazoline analogs of doxorubicin (O-DOX) and daunorubicin (O-DAU) to induce apoptosis and autophagy in ovarian and liver cancer cells. Reactive oxygen and nitrogen species (ROS and RNS, respectively), together with intracellular calcium-mediated downstream signaling, are essential for the anticancer effect of these new anthracycline analogs. The changes of mitochondrial membrane potential and induction of the ceramide pathway suggests that these compounds induce cell death by apoptosis. In addition, a significant increase of autophagosome formation was observed by fluorescence assay and acridine orange staining, indicating that the new analogs also induce autophagic cell death. Compared to free DOX- and DAU-treated cells, we observed inhibition of colony formation and migration, a time-dependency between ROS/RNS levels and a greater fall in mitochondrial membrane potential. Altogether, our research broadens the base of molecular oxazolinoanthracyclines targets and reveals that derivatives mediated oxidative stress, ceramide production and increase in intracellular calcium level by mitochondria. Furthermore, our data highlight the importance of mitochondria that simultaneously assume the role of activator of autophagy and apoptosis signals.

## Introduction

Anthracycline antibiotics are anti-neoplastic drugs that are effective against both hematological malignancies and solid tumors [1]. The mechanisms of action of doxorubicin (DOX) and daunorubicin (DAU) have been associated with DNA damage, topoisomerase inhibition and reduction in the presence of free iron [2]. There is an urgent need for new approaches to anthracycline chemotherapy that could improve therapeutic index and overcome drug resistance, for example, by specific modification of parent drug structures. We modified DOX and DAU structures by creating an oxazoline ring at the daunosamine moiety through introduction of a NH_2_ group at the C-3′ position of the daunosamine moiety. Chemical modification leading to the oxazolinoanthracycline structures, increased their cytotoxic ability to overcome the drug-resistance barrier. O-DOX was active against cell lines with different resistance phenotypes, including those with high expression of P-gp and MRP1 genes: MES-SA, MES-SA/DX (DOX-resistant variant), LoVo, LoVo/DX (DOX-resistant variant), HL-60, HL-60/MX2 (mitoxantrone-resistant variant) and HL-60/Vinc (vincristine-resistant variant) cell lines [3, 4]. Studies on their mechanism of action will allow us to develop more effective chemotherapy strategies.

Reactive oxygen and nitrogen species generated by anthracyclines have drawn attention as novel signal mediators that are involved in growth, differentiation, progression and death of cancer cells [5]. In addition, calcium and ceramide contribute to a wide variety of intracellular signaling pathways as second messengers [6, 7]. Here we have studied the roles of stress responses from mitochondria, generated by new chemotherapeutics in solid tumor cells, which have been shown to function as a platform for apoptotic or autophagic signaling. Previously we confirm genotoxic properties of compounds, the ability to induce apoptosis through the mitochondrial pathway by measure mRNA expression levels of the genes encoding PARP-1 (*PARP1*), caspase-3 (*CASP3*), caspase-9 (*CASP9*), Bcl-2 (*BCL2*), survivin (*BIRC5*), histone H2AX (*H2AFX*), and cytochrome *c* (*CYC2*), by analysis of cell death type by fluorescence double-staining or by externalization of phosphatidylserine. Moreover, we also evaluated the intracellular accumulation and uptake of the derivatives, effects on the cell cycle and proteasome 20S activity.

Most cell death is considered to occur via necrotic and apoptotic processes. However, other forms of programmed cell death may be considered, such as autophagy. This is a process of selective degradation of cellular components [8]. Many studies have shown an interplay between apoptosis and autophagy and have indicated that induction of apoptosis is often related to autophagy. Similarly, in addition to the intrinsic, mitochondrial-mediated, and the extrinsic, receptor-mediated, pathways, new mechanisms of induction of apoptosis have been discovered, including the ceramide-induced pathway. Current information concerning transduction of antiproliferative and death stimuli in cells permits understanding of the mechanisms of action of known drugs and also suggests novel therapeutic targets for treatment of diseases such as cancer [9].

Among the five sphingomyelinases, the lysosomal acidic sphingomyelinase (SMase) and the magnesium-dependent neutral sphingomyelinase (nSMase) are considered the main candidates for production of ceramide in the cellular response to stress. Ceramide is a bioactive sphingolipid that has been implicated in numerous biological processes, such as cell cycle arrest, apoptosis, senescence and autophagy [10]. Ceramide can be generated by de novo synthesis, salvage of sphingosine, or hydrolysis of sphingomyelin. Activation of nSMase occurs through different mechanisms, including post-translational activation via phosphorylation on five serine residues in response to oxidative stress through a p38 mitogen-activated protein kinase mechanism. Moreover, upregulation of nSMase has been shown to be mediated by Sp1 and Sp3 transcription factors through direct binding to the nSMase promoter in response to all-trans retinoic acid (ATRA) and daunorubicin [11]. DOX is involved in the synthesis of ceramide, followed by activation of the CREB3L1 transcription factor [12]. However, the precise mechanisms for generation of ceramide and its regulation remain unknown.

The aim of the present study was to determine biological properties of oxazolinoanthracyclines, such as ability to generate of oxidative/nitrosative stress, changes in mitochondrial membrane potential or in intracellular calcium level. We would like to confirm the role of the mitochondrion in the process of apoptosis and indicate the next points of action of the tested derivatives like neutral sphingomyelinase. We are looking for new molecular target of DOX and DAU derivatives. In addition, we have expanded the research not only to look for new proteins involved in the process of apoptosis, but also to check whether anthracycline derivatives can induce other types of cell death as for example autophagy.

## Materials and methods

### Reagents

Acridine orange, 2,3-diaminonaphthalene (DAN), 5,5′,6,6′-tetrachloro-1,1′,3,3′-tetraethyl-benzimidazol-carbocyanine iodide (JC-1), 2′,7′-dichlorodihydrofluorescein diacetate (DCFH_2_-DA), and Autophagy Assay kit were obtained from Sigma Aldrich cat. no.:MAK138 (St. Louis, Missouri, USA). Human MAP1LC3A (Microtubule-associated proteins1A/1B light chain 3A) ELISA Kit was obtained from Fine-test (Wuhan, Hubei, China). The Fluo-4 NW Calcium Assay Kit was obtained from Molecular Probes (Eugene, USA). The Sphingomyelinase Assay kit was purchased from Abcam (Cambridge, UK). Culture media (DMEM, RPMI 1640) and fetal bovine serum (FBS) were obtained from Cambrex (Basel, Switzerland); trypsin-EDTA, penicillin and streptomycin were acquired from Sigma Aldrich (St. Louis, Missouri, USA).

### Cell culture and drug administration

**Fig 1:**
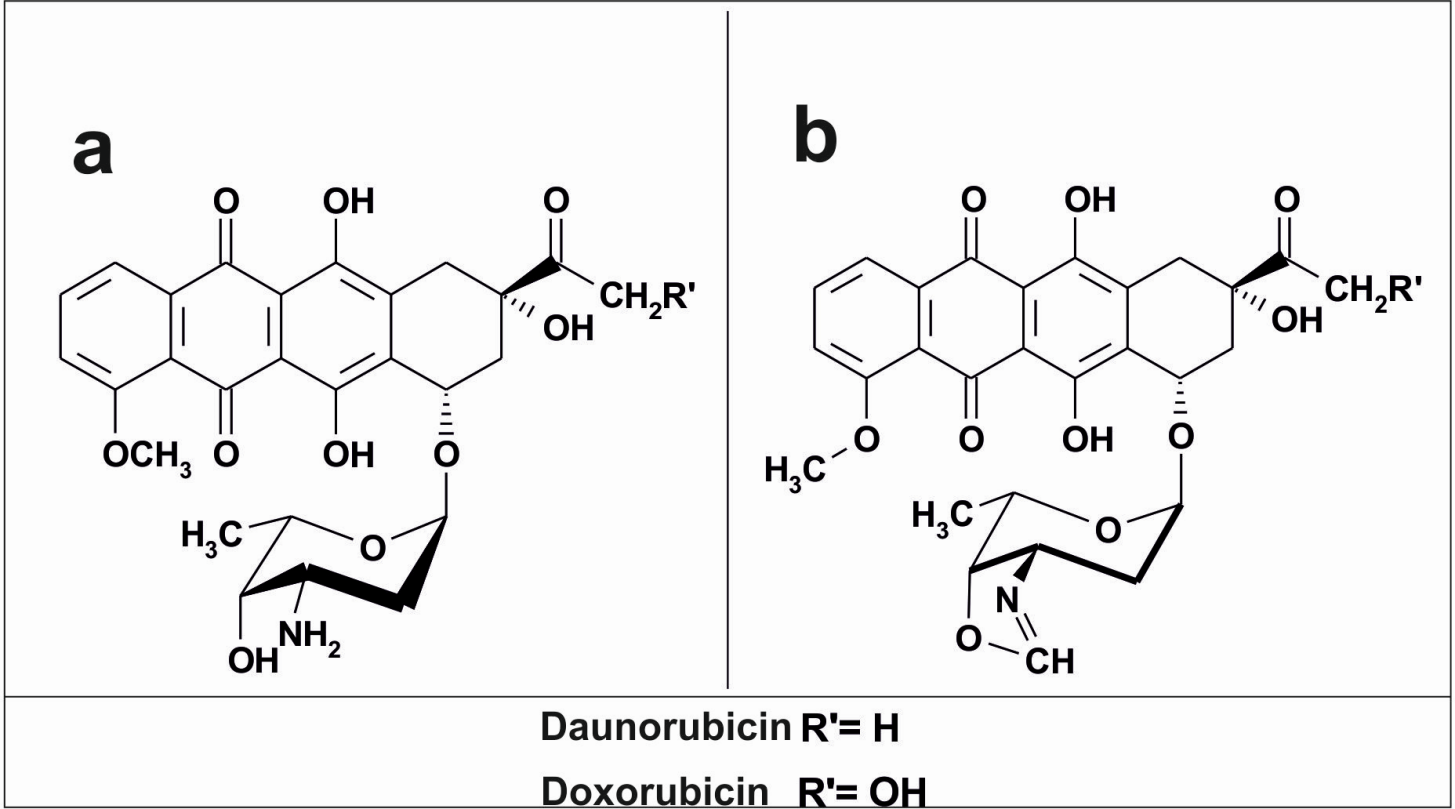
Chemical structures of (a) doxorubicin, daunorubicin; (b) oxazolinodoxorubicin and oxazolinodaunorubicin.

The human HepG2 (*human hepatocellular carcinoma*, ATCC HB-8065) and SKOV-3 (*human ovarian adenocarcinoma*, ATCC HTB-77) cell lines used in the experiments were received from the American Type Culture Collection (ATCC; Rockville, MD, USA). The cell lines were obtained between 2010 and 2013. The newly received cells were expanded and aliquots of less than 10 passages were stored in liquid nitrogen. All cell lines were kept at low passage, returning to original frozen stocks every 6 months. During the course of this study, cells were thawed and passaged within 2 months in each experiment. The cells were cultured in DMEM and RPMI with 10% FBS, penicillin (10 U/mL) and streptomycin (50 μg/mL) and regularly checked for mycoplasma contamination. The cells were cultured under an atmosphere of 5% CO_2_ and 95% air at 37°C. The IC_50_ values of the tested compounds were determined previously in SKOV-3 and HepG2 cells and listed in the Table 1 [13]. Due to the observed toxic effects, the following experiments were performed with the use of anthracyclines and their oxazoline derivatives in a concentration of 80 nM. In some experiments, a portion of the cells were preincubated with an antioxidant (1 mM NAC) for 1 h, and then the drugs were added and incubation continued for the required period of time under the same conditions. Doxorubicin (DOX), daunorubicin (DAU) and their oxazoline derivatives, designated as O-DOX (oxazolinodoxorubicin, 3′-deamino-3′-N, 4′-O-methylidenodoxorubicin) and O-DAU (oxazolinodaunorubicin, 3′-deamino-3′-N, 4′-O-methylidenodaunorubicin), were synthesized at the Institute of Biotechnology and Antibiotics (Warsaw, Poland) (Fig 1). Purity of all compounds was ≥ 98.5% according to HPLC method [14]. Oxazolinoanthracyclines was dissolved in the mixtures: DMSO/water 1:1 v/v. PBS was used for further dilutions. They were stored frozen at −20°C divided into small portions. Concentrated drug solutions were thawed immediately before use, then diluted in PBS and added to the cell medium at the final concentration. Induction of apoptosis and autophagy in the presence or absence of the antioxidant, N-acetylcysteine (NAC), or the autophagy inhibitor, 3-methyladenine (3-MA), was also investigated. We chose NAC as an antioxidant because of its ability to enhance the natural antioxidant defense by increasing intracellular concentrations of reduced glutathione (GSH) [15]. NAC has proven efficacy for attenuating oxidative stress and improving eNOS/NO (endothelial nitric oxide synthase) signaling [16, 17].

**Table 1:** The value of IC_50_ parameters for DOX and O-DOX, or DAU and O-DAU after 72 h incubation.

Compound	Cell lines (IC_50_ ± SD)	
	SKOV-3 [nM]	Hep-G2 [nM]
DOX	346.74 ± 35	529.65 ± 51
O-DOX	67.29 ± 12	75.05 ± 6
DAU	302.59 ± 64	392.81 ± 10
O-DAU	295.83 ± 10	85.45 ± 19

### Measurements of ROS production

Production of ROS was measured using the DCFH_2_-DA probe [18, 19]. Intracellular ROS levels were determined directly in cell monolayers in 96-well microplates using a Fluoroskan Ascent FL microplate reader (Labsystems, Stockholm, Sweden). Cells were preincubated with DCFH_2_-DA in the culture medium at a final concentration of 5 μM for 30 min at 37°C [20]. The kinetics of ROS generation in SKOV-3 and HepG2 cells after treatment with an 80 nM concentration of drugs were measured up to 4 h in the presence or absence of an antioxidant. In our experiments, we used 500 μM of H_2_O_2_ (as a positive control) which increases oxidative stress by generation of ROS [21, 22]. The fluorescence of DCF was measured at 530 nm after excitation at 485 nm (DCFH_2_-DA, after deacetylation to DCFH_2_, is oxidized intracellularly to its fluorescent derivative, DCF). Assays were performed in modified Hank’s buffered salt solution (HBSS) (140 mM NaCl, 5 mM KCl, 0.8 mM MgCl_2_, 1.8 mM CaCl_2_, 1 mM Na_2_HPO_4_, 10 mM HEPES and 1% glucose, pH 7.0, without phenol red).

### Nitrite determination

Nitrite levels were determined using the fluorescent probe 2,3-diaminonaphthalene. DAN reacts with nitrite under acidic conditions to form fluorescent 1-(H)-naphthotriazole. NO donor-sodium nitroprusside (SNP; 100, 300 μmol/L) was used as positive control (data unpublished). Intracellular nitrite levels were determined directly in cell monolayers in 96-well microplates using a Fluoroskan Cary Eclipse. The fluorescence was measured at 415 nm after excitation at 365 nm. The cells were plated in 96-well black plates (10^4^ cells/well) and treated with DOX, DAU, O-DOX or O-DAU. The incubations were carried out with drugs for different times (0.5–2 h). Then 100 μl medium was tranfered into a 96-microwell plate. In the next step 50 μL of freshly prepared DAN (0.025 mg/mL in 0.62 N HCl) and 50 μL of 1.5 N HCl were added to each well and mixed immediately. After 5 min incubation at 30°C in the darkness, the reaction was terminated with 7 μL of 3 N NaOH.

### Intracellular calcium assay

Intracellular calcium levels were determined using the fluorescent probe Fluo-4 NW. The cells were plated in 96-well black plates (10^4^ cells/well) and treated with DOX, DAU, O-DOX or O-DAU. The incubations were carried out with drugs for different times (0–48 h). The medium was then removed and the cells were washed with sodium phosphate buffered saline (PBS). Finally, a dye loading solution at concentration of 5 μM was added at a volume of 100 μL per well. The measurements were calculated on a fluorescence plate reader for excitation at 494 nm and emission at 516 nm (Fluoroskan Cary Eclipse).

### Mitochondrial membrane potential (ΔΨ_m_)

Cells were seeded into 96-well microplates. After 24 h, 80 nM concentrations of the compounds were added to the wells. The cells were incubated with the drugs for 2, 4, 24, and 48 h. At the end of treatment, the medium was removed and the cells were incubated in total darkness with 5 μM JC-1 in HBSS for 30 min at 37°C. The fluorescence of both JC-1 monomers and dimers was measured on a Fluoroskan Ascent FL microplate reader using filter pairs of 530/590 nm (dimers) and 485/538 nm (monomers). Prior to fluorescence measurements and photography, cells were washed twice with HBSS to remove the dye, which otherwise could have adsorbed on the microplate well plastic and distorted measurements. The results given in the figures are shown as a ratio of dimer to monomer fluorescence in relation to the control fluorescence ratio, taken as 100%. The cells presented in the images were incubated with drugs for 48 h. JC-1 fluorescence incidence was photographed immediately after drug treatment with an inverted Olympus IX70 fluorescence microscope (Olympus, Tokyo, Japan).

#### Autophagy detection

The Autophagy Assay kit provides a simple and direct procedure for measuring autophagy in a variety of cell types using a proprietary fluorescent autophagosome marker. The ability of the investigated compounds to induce autophagy was measured in accordance with the manufacturer’s protocol. Cells were plated in 96-well black fluorimetric plates (10^4^ cells/well) and then treated with DOX, O-DOX, DAU or O-DAU. The autophagy inhibitor (3-MA, 3-methyladenine) was used in the control experiments to confirm that the observed fluorescence in the drug-treated cells was due to autophagosomes present in the samples. After incubation (4, 24, 48 h) the growth medium was removed and the cells were washed with PBS to eliminate sources of baseline fluorescence. Finally, the autophagosome detection reagent working solution (prepared in accordance with the manufacturer’s protocol) was added at a volume of 100 μL per well and incubated for 30 min in total darkness at 37°C. After incubation with stain solution, the cells were washed four times by gently adding 100 μL of wash buffer to each well. Measurements were conducted on a Fluoroskan Ascent FL microplate reader using 355 nm excitation and 538 nm emission wavelengths and visualized under a fluorescence microscope (Olympus IX70).

## Acridine orange staining

Acridine orange is a cell-permeable cationic dye that enters acidic compartments such as lysosomes. The dye emits orange light under low pH conditions, thus it is often used to monitor changes in autophagy. It crosses into lysosomes (and other acidic compartments), becoming protonated. Outside acidic compartments, acridine orange emits green fluorescence. Briefly, SKOV-3 and HepG2 cells were plated in 30 mm Petri dishes (10^5^ cells/well) and then treated with DOX, O-DOX, DAU or O-DAU. At the indicated time points, cells were washed with PBS and put in a trough with acridine orange working solution (2 mg/mL). After 5 min of staining, the dishes were washed gently with PBS and then examined under a fluorescent microscope (Olympus IX70, Japan).

### Autophagic detection with LC-3 assay

LC-3 assay was carried out in accordance with the manufacturer’s protocol. Cells were seeded into cell culture Petri dishes at a density of 5 x 10^5^ and cultured with tested compounds up to 48 h. After drugs treatment, the cells were washed with PBS and re-suspended in an ice-cold cytosol extraction buffer containing 1 mM phenylmethylsulfonyl fluoride (PMSF) and a protease inhibitor cocktail. Thus, obtained cell lysates were centrifuged at 10000 *g* for 30 min at 4°C. The protein concentration was determined by using the Bradford method. The supernatants (cytosolic fraction) were collected and stored at −80°C. AntiMAP1LC3 antibody was pre-coated onto 96-well plates. The clarified cytoplasm extracts, LC-3 standard and blank control were added to the wells, and incubated for 90 min at 37°C. In the next step, biotin conjugated antiMAP1LC3A antibody working solution was added into each well, and reactions were continued for 1 h at 37°C. Immediately after the incubation period, HRP-Streptavidin working solution was added (30 min, 37°C) and unbound conjugates were washed away with wash buffer. The absorbance of light at 450 nm was proportional to the MAP1LC3 (Microtubule-associated proteins 1A/1B light chain 3A) amount of sample captured in plate. The plates were measured using a microplate reader (BioTek, Winooski, VT, USA).

### Sphingomyelinase assay

Neutral sphingomyelinase activity was measured in accordance with the manufacturer’s protocol. The clarified cytoplasm extracts (obtained as in LC-3 assay), sphingomyelinase standards and blank control were added to the wells to determine the cellular level of sphingomyelinase. In the next step sphingomyelin working solution was added into each well, and reactions were continued for 1 h at 37°C. Additionally, sphingomyelinase assay mixture was added into each well and cells were incubated for 1 h at room temperature (protected from light). AbBlue indicator was then used as a colorimetric probe to indirectly quantify the phosphocholine produced by the SMase-catalyzed hydrolysis of sphingomyelin (SM). The absorbance of light at 655 nm was proportional to the formation of phosphocholine, and therefore to the SMase activity. The plates were measured using a microplate reader (BioTek, Winooski, VT, USA).

### Clonogenic assay

The effect of DOX, DAU and oxazoline derivatives on cell growth was assessed using a clonogenic assay. For this analysis, 200 cells (HepG2 and SKOV-3 cell line) were plated onto six-well plates in growth medium and after overnight attachment cells were exposed to 80 nM concentrations of drugs for 4 or 48 h. The cells were then washed with medium and allowed to grow for 14 days under drugs-free conditions, after which the cell colonies were fixed with methanol mixed with acetic acid (7:1) for 10 min and stained with 0,5% crystal violet for 20 min. The plates were rinsed with water, air-dried, photographed and evaluated for colony estimation. Colonies containing more than 50 cells were counted. All experiments were performed in triplicate.

### Cell migration assay

Migration was measured by wound healing assay, in which cells were grown to 80% confluence in 6-well plates, streaked with a sterile 1000 μl pipette tip, and allowed to recover in DOX, ODOX, DAU and ODAU-treated media. Images of the scratches were captured from 0 to 75 h in SKOV- cells and from 0 to 55 h in HepG2 cells. Cells were visualized at 10 magnification and migration determined by measuring wound width (in nm) using cellSens Dimension imaging software (Olympus IX70, Tokyo, Japan).

### Statistical analysis

Data were presented as a mean ± standard deviation (SD). Multiple comparisons were made using analysis of variance with Tukey’s *post hoc* test. All analyses were performed using the STATISTICA program (StatSoft, Tulsa, USA). A *p*-value of < 0.05 was considered significant. Significant differences are indicated in the figures as follows: * *p* < 0.05 compared with control cells (taken as 100%); + *p* < 0.05 between samples preincubated for 1 h with 1 mM NAC and then incubated with test compounds; # *p* < 0.05 between samples incubated with DOX and O-DOX or DAU and O-DAU. All values are given as the mean ± SD of three independent experiments.

## Results

### The oxazolinoanthracyclines produce less ROS than the parent compounds

**Fig 2:**
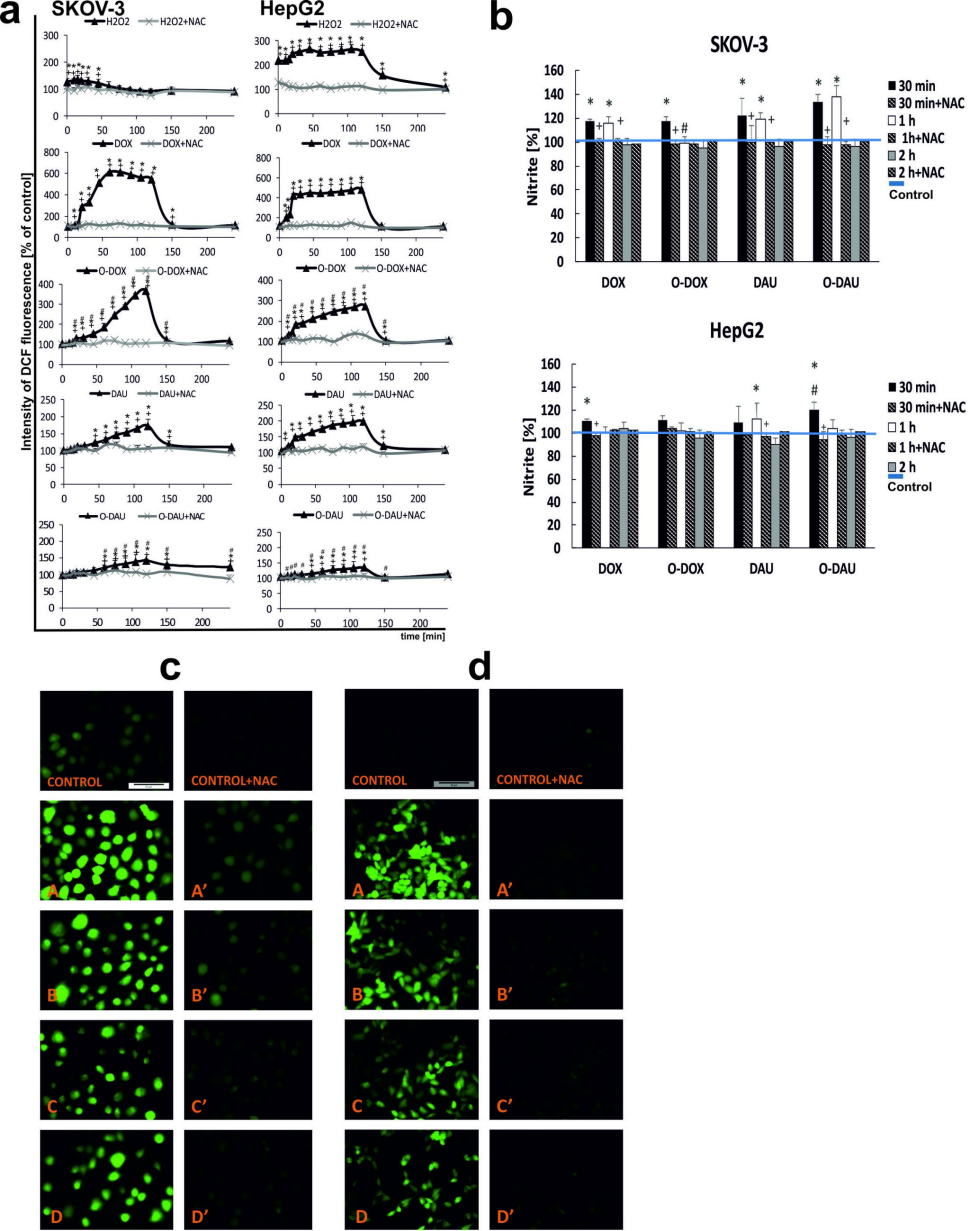
Oxazolines induce oxidative stress. (**a**) The effect of drugs on ROS production in SKOV-3 and HepG2 cells. (**b**) The effect of drugs on nitrite level. (**c**) SKOV-3 cells and (**d**) HepG2 cells stained with the fluorescence probe H_2_DFC-DA after 1 h incubation with 80 nM concentrations of investigated compounds: DOX (A), O-DOX (B), DAU (C), O-DAU (D). In experiments with the antioxidant, cells were preincubated with 1 mM NAC for 1 h. Then DOX, DAU or oxazoline derivatives (80 nM) were added and incubation was continued for up to 4 h (scale bar: 50 μm): DOX (A′), O-DOX (B′), DAU (C′), O-DAU (D′).

ROS production was examined as an index of oxidative stress during incubation of SKOV-3 and HepG2 cells with DOX, DAU and their oxazoline derivatives in the presence or absence of NAC using the fluorescence probe DCFH_2_-DA. Fig 2A shows quantitative data expressed as a percentage of DCF fluorescence intensity in cells incubated with the drugs up to 4 h. The kinetics of ROS formation were time-dependent in both cell lines. Moreover, ROS production did not seem to be directly related to the sensitivity of a particular cell line to anthracyclines. Our results clearly demonstrate that slightly basic compounds like DOX and DAU generate higher levels of ROS in SKOV-3 cells than their oxazoline derivatives (Fig 2A). Maximal increases in ROS levels were observed after 2 h treatment of cells with DOX (546%) and DAU (172%). At the same time point, ROS levels were increased by 365% with O-DOX and 142% with O-DAU versus control, untreated cells. A similar pattern was observed in HepG2 treated cells. DOX and DAU increased ROS levels by 481% and 197%, respectively. In contrast, the changes observed after treatment with O-DOX and O-DAU were significantly lower. After 2 h incubation with the investigated compounds, the ROS levels decreased significantly and approached the level of the control when incubation was extended to 4 h.

When the cells were preincubated with N-acetylcysteine, ROS levels were significantly decreased compared to the control probes for all tested incubation times. The early increase in ROS formation observed in this work after treatment the cells with oxazoline derivatives, albeit significantly lower than after treatment with the parent compounds, suggests that ROS generation is an important mechanism of action that might contribute to the cytotoxicity of new oxazolinoanthracycline in liver and ovarian cancer cells. Microscopic examination of cells stained with H_2_DCF-DA revealed increased DCF fluorescence in drug treated cells resulting from ROS-mediated oxidation of the probe (Fig 2C and 2D).

### The reactive nitrogen species are produced after treatment with oxazolinoanthracyclines

Nitric oxide reacts with molecular oxygen and water to form nitrite. The nitrite detection protocol used in this study was about 50 times more sensitive than the commonly used Griess reaction [23]. It also permitted the study of minimal changes in the NO pathway. Nitrite reacts with 2,3-diaminonaphthalene (DAN) to form the fluorescence [23].

Fig 2B shows quantitative data expressed as a percentage of 2,3-naphthotriazole fluorescence intensity in cells incubated with the drugs for 30 min, 1 h and 2 h. We observed early changes in nitrite levels after treatment with oxazoline derivatives. Doxorubicin and O-DOX in SKOV-3 and HepG2 cells generated similar levels of reactive nitrogen species after 30 min incubation (117% and 111%, respectively). The highest RNS level was generated in SKOV-3 cells with O-DAU (133%), while incubation with DAU led to a level of 122%. The RNS changes in HepG2 cells were smaller, at indicated time point, but had a similar trend, with O-DAU also producing the greatest change (120%).

The RNS level was time-dependent and considerably greater in cells incubated with O-DAU, reaching a maximum at 1 h of incubation (138%) in SKOV-3 cells. The RNS level after DOX treatment decreased slightly in SKOV-3 cells, whereas DOX and O-DOX gave control levels in HepG2 cells. After prolonging the incubation to 2 h, RNS were undetectable.

Our results clearly demonstrate that oxazolines are able to generate RNS in cancer cells derived from solid tumors. In both cell lines, almost complete inhibition of RNS production was observed in cells pretreated with NAC.

### Early changes in intracellular calcium levels generated by O-DOX and O-DAU

The levels of cytoplasmic calcium were determined using the fluorescence probe Fluo-4 NW. In both cell lines treated with anthracyclines we detected early changes in calcium levels. At the initial time point, 2 h of SKOV-3 incubation, there was no difference between DOX (213%) and O-DOX (206%) action. Differences in the change of intracellular calcium levels were only observed after 2 h treatment with DAU and O-DAU despite the lack of differences in the cytotoxicity of drugs in the SKOV-3 line. Initially O-DAU produced greater changes than DAU. This trend reversed after a longer incubation time of 4 h, after which DAU led to higher calcium levels. The greatest changes in intracellular calcium level were observed after 4 h O-DOX treatment, which increased levels by 279% versus control, untreated cells.

**Fig 3:**
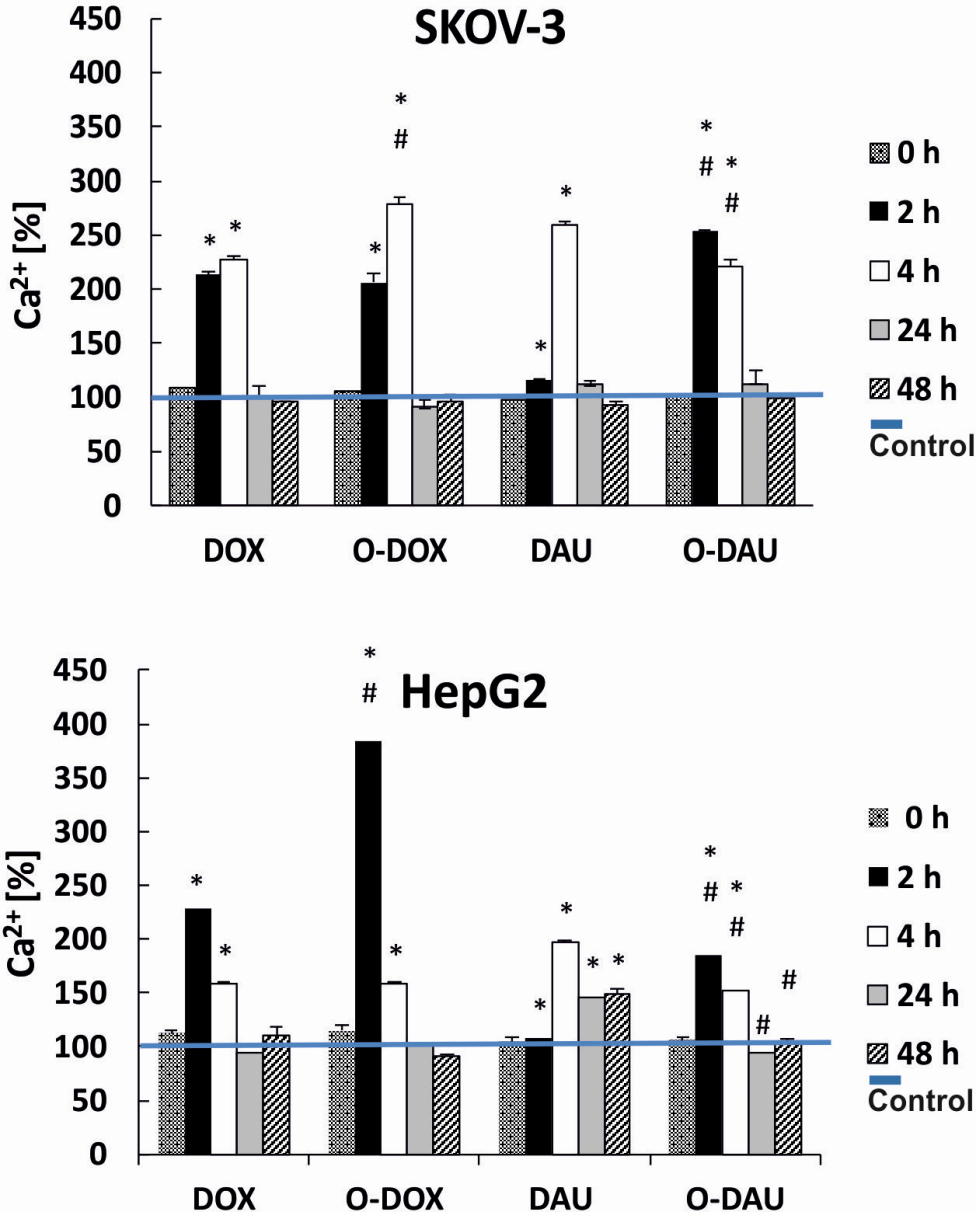
Influence of drugs on intracellular levels of Ca^2+^ in SKOV-3 and HepG2 cells. The cells were treated with 80 nM concentrations of drugs for 2, 4, 24 or 48 h. In experiments with antioxidant, cells were preincubated with 1 mM NAC, then drugs were added and incubation continued for another 2–48 h.

In liver cancer cells, higher levels of intracellular calcium were induced by both oxazoline derivatives compared to DOX and DAU after 30 min incubation. Increasing the incubation time decreased the level of intracellular calcium. A delay in this process was again observed after treatment with DAU, which was the only drug to generate a positive increase after 4 h incubation. Further extending the duration of drug treatment led to significant declines in the levels of calcium ions (Fig 3).

### Oxazolinoanthracyclines reduced the mitochondrial membrane potential (ΔΨ_m_)

**Fig 4:**
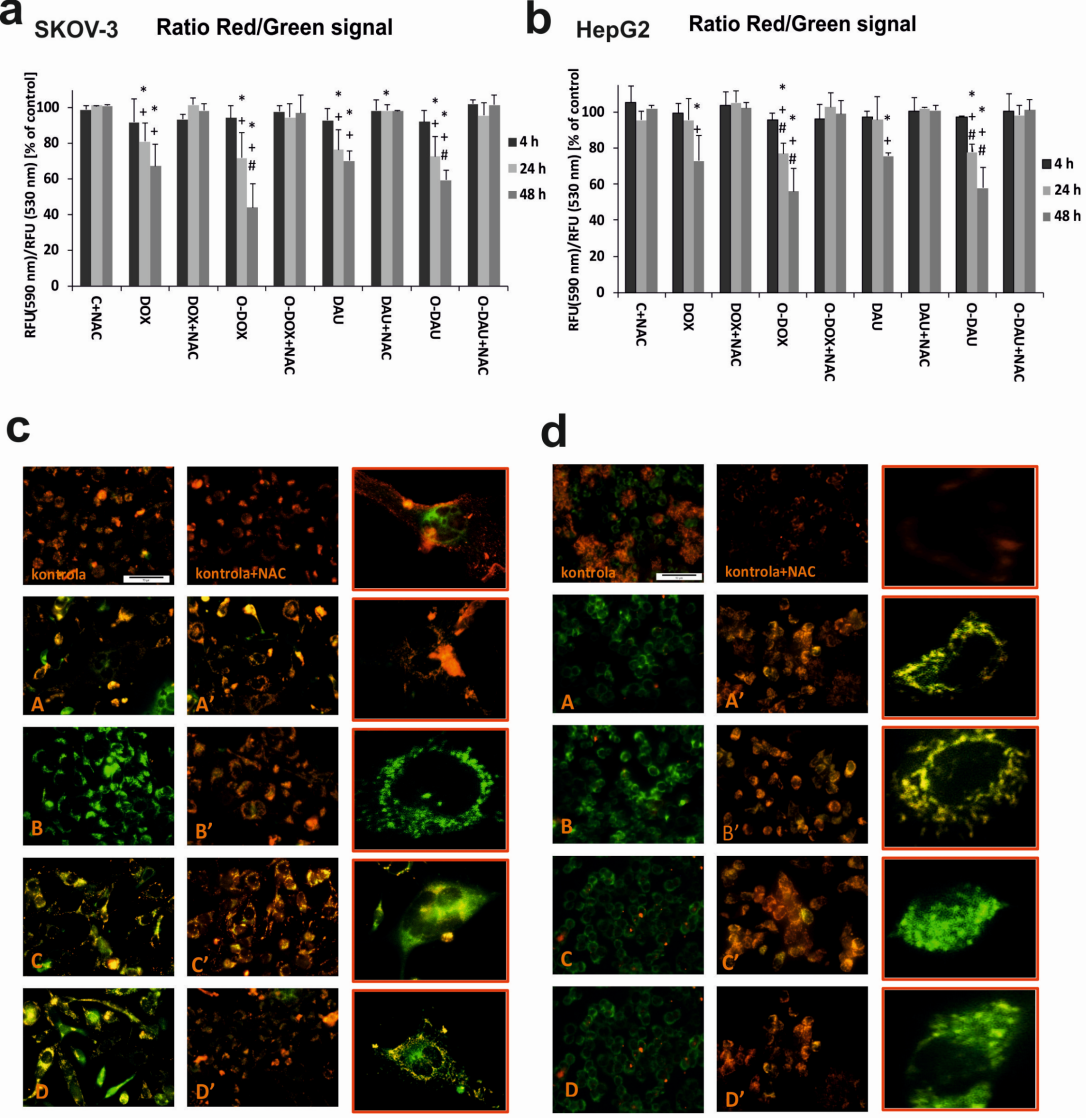
Oxazolinoanthracyclines reduced the mitochondrial membrane potential. Changes in mitochondrial membrane potential of (**a**) SKOV-3 and (**b**) HepG2 cancer cells. The fluorescence ratio of JC-1 dimers/JC-1 monomers in the control was assumed to be 100%. (**c**) SKOV-3 cells and (**d**) HepG2 cells stained with the fluorescence probe JC-1 after 48 h incubation with 80 nM concentrations of investigated compounds: DOX (A), O-DOX (B), DAU (C), O-DAU (D). In the experiments with antioxidant, the cells were preincubated with 1 mM NAC for 1 h, then DOX, DAU or oxazoline derivatives (80 nM) were added and incubation were continued for up to 48 h (scale bar: 50 μm): DOX (A′), O-DOX (B′), DAU (C′), O-DAU (D′). The right-hand column shows enlarged representative images of single drugs treated cells, scale bar: 10 μm. The cells were stained with JC-1 probe. Yellow-orange fluorescence of JC-1 dimers is present in cell areas with high mitochondrial membrane potential, while green fluorescence of JC-monomers is prevalent in cell areas with low mitochondrial membrane potential. The cells were visualized under an inverted fluorescence microscope (Olympus IX70, Tokyo, Japan). (For interpretation of the colours, the reader is referred to the web version of the article).

The impact of chemical modification of the anthracycline structure on mitochondrial membrane potential (ΔΨ_m_) in SKOV-3 and HepG2 cells was assessed using fluorimetric analysis after staining with the fluorescent dye JC-1. Fig 4C and 4D show the accumulation of JC-1 within active mitochondria of SKOV-3 and HepG2 cells after exposure to modified and unmodified anthracyclines. The red fluorescence of JC-1 dimers (with a high mitochondrial potential) was observed in control cells. In drug-treated cells, a remarkable increase in the green fluorescence of JC-1 monomers was seen, indicating reduced mitochondrial membrane potential (Fig 4A and 4B). The most intense green fluorescence was noted after treatment of SKOV-3 cells with O-DOX. In HepG2 cells, strong green fluorescence of JC-1 monomers was observed after treatment with both O-DOX and O-DAU. In probes preincubated with antioxidant, the fluorescence observed in test cells was similar to that in control cells (quantitative data are shown in Fig 4A and 4B). All drugs induced time-dependent changes. After 4 h incubation, the differences between the drugs were not noticeable. The ratio of JC-1 probe fluorescence in SKOV-3 cells decreased by about 20%–30% at 24 h after treatment with drugs. At this time, there were no differences between the effects of DOX or DAU and O-DOX or O-DAU. Differences did not appear until 48 h of incubation, when O-DOX caused a 24% greater decrease in mitochondrial membrane potential than DOX, and O-DAU caused a 12% greater decrease than DAU.

In HepG2 cells, changes in ΔΨ_m_ were only observed after 24 h of incubation with the new anthracyclines. Only extension of incubation for 48 h gave a drop in mitochondrial membrane potential of approximately 56% for both oxazoline derivatives. The difference between DOX and O-DOX was 16%, and between O-DAU and DAU was 18%. When the samples were preincubated with the antioxidant NAC, the mitochondrial membrane potential was similar to the control. Our results suggest that changes in ΔΨm were dependent on both drug concentration and the time of drugs treatment. In the SKOV-3-line, collapse of ΔΨm was correlated with the sensitivity of the cell line to drugs. On the other hand, in the HepG2 cell line, O-DOU modified membrane potential even more despite the lack of difference in the cytotoxicity DAU and O-DAU.

### Modified anthracyclines are able to trigger autophagy but to a lesser degree than unmodified drugs

**Fig 5:**
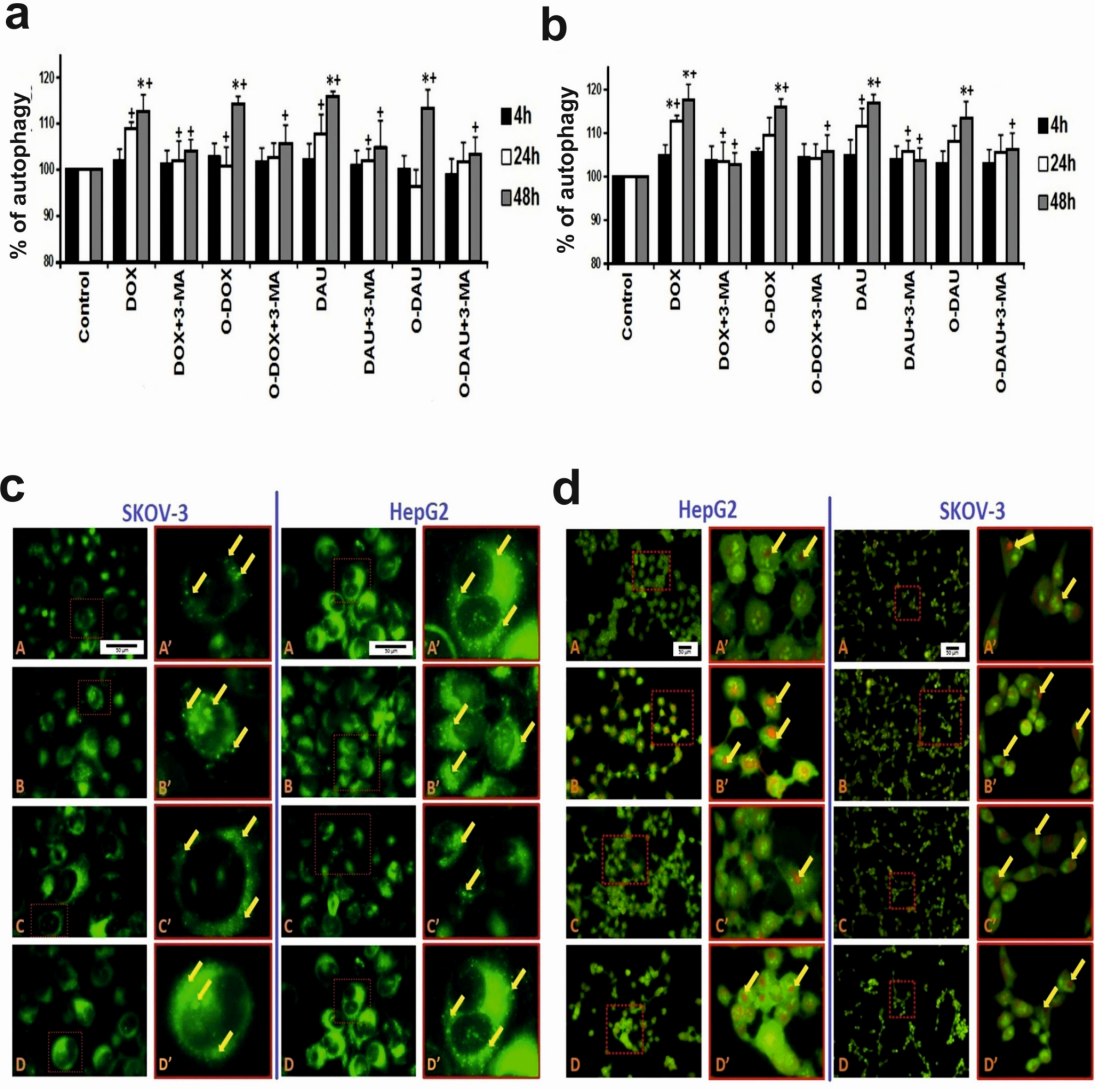
Modified anthracyclines are able to trigger autophagy. Fraction of autophagic cells in (**a**) SKOV-3 and (**b**) HepG2 cancer cell lines. The measurements were carried out in the presence or absence of the inhibitors. The cells were treated with 80 nM concentrations of DOX, O-DOX, DAU or O-DAU and then incubated for 4, 24 or 48 h. Autophagic changes in control cells (without drug treatment) measured after 2, 4, 24 and 48 h incubation were taken as 100%. 3-MA (5 mM) was added to SKOV-3 or HepG2 cell lines in the presence of 80 nM DOX, O-DOX, DAU or O-DAU for a period of 4, 24 and 48 h. (+) p < 0.05—statistically significant differences observed between the investigated probes in comparison to the effect after treatment with the 3-MA inhibitor. (**c**) Fluorescence images of autophagic SKOV-3 and HepG2 cells after 48 h incubation with 80 nM concentrations of investigated compounds. The left-hand column shows images of cells treated with DOX (A), O-DOX (B), DAU (C), O-DAU (D). Some cells display abnormal morphology: giant cells and elongated cells with protrusions from the plasma membrane (blebbing); cytoplasmic bridges between cells. The right-hand column shows representatives images of single cells with progressive autophagosome formation (autophagy is indicated by bright green staining of autophagic vacuoles–marked by the yellow arrows). (**d**) Fluorescence images of SKOV-3 and HepG2 cells after 48 h incubation with 80 nM concentrations of investigated compounds. Cells were stained with acridine orange (AO). The left-hand column shows images of cells treated with DOX (A), O-DOX (B), DAU (C), O-DAU (D). The right-hand column shows representative images of single cells with progressive autophagosome formation (autophagy is indicated by bright red staining of autophagosomes–marked by the yellow arrows). (**c, d**) Cells were analyzed with an inverted fluorescence microscope (Olympus IX70, Tokyo, Japan). Scale bar: 50 μm.

The results show that unmodified (DOX and DAU) and modified (O-DOX and O-DAU) anthracyclines are able to trigger autophagy of SKOV-3 and HepG2 cells (Fig 5A and 5B). The percentage of autophagic cells was positively correlated with incubation time in the presence of the investigated compounds. Slightly greater changes were observed after treatment with unmodified anthracyclines using a fluorescent method based on the detection of autophagosomes. The highest percentage of autophagic cells was found after 48 h treatment with DOX (SKOV-3, 114%; HepG2, 118%) and DAU (SKOV-3, 116%; HepG2, 117%). During the 4 h post-incubation period, no significant changes in the autophagic cell fraction were observed after exposure to all investigated compounds. Administration of the autophagy inhibitor, 3-MA, decreased autophagic cell death in all investigated time periods (Fig 5A and 5B).

Visualization of cells (after 48 h treatment with investigated compounds) by fluorescence microscopy revealed not only autophagosome formation, but also abnormal morphology, which included giant cells, elongated cells with protrusions from the plasma membrane (blebbing), cytoplasmic bridges between cells and chromatin condensation. Autophagy is indicated by bright green dot staining of autophagic vacuoles (Fig 5C and 5D).

The information obtained by using the fluorescence experimental techniques were supported and confirmed by a high sensitive, immuno-enzymatic investigation based on ELISA method [24]. The obtained data clearly indicate that the percentage of autophagic related proteins was positively correlated with incubation time. Treatment with the modified drugs generated a lower level of LC3 protein in comparison to DOX, as well as DAU-treated cultures, especially after longer incubation times. It should be also taken into account that during the 48h treatment period, the percentage of the LC3 protein increased in comparison to 24h incubation time after all investigated compounds treatment. In SKOV-3 cancer cells, after 48h of treatment with the tested compounds, the maximum level of LC3 protein was observed after DAU exposure (~145%). A slightly lower level of autophagic protein was also noted after treatment with DOX (~133%). O-DOX and O-DAU, at this time point, were the least potent in inducing autophagosomes formation. Similar effect was observed in HepG2 cell line, where DOX and DAU caused LC3 protein content at the level of ~128% and 154% in comparison to O-DOX (~120%) and O-DAU (~129%).

**Fig 6:**
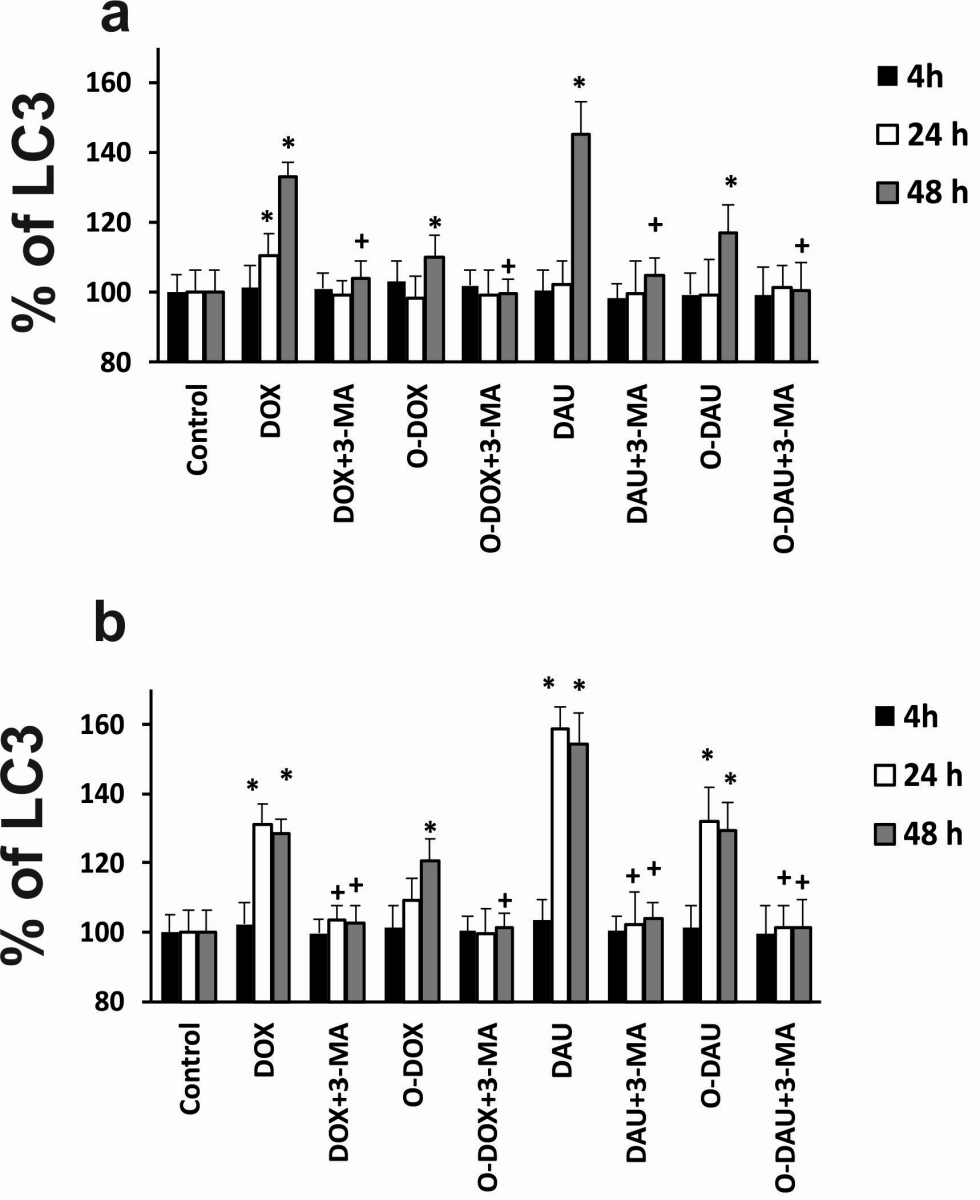
LC3 protein contents after DOX, DAU and oxazolinoanthracyclines treatment in the SKOV-3 and HepG2 cells. The measurements were carried out in the presence or absence of the inhibitor, 3-MA (5 mM). (+) p < 0.05—statistically significant differences observed between the investigated probes in comparison to the effect after treatment with the 3-MA inhibitor.

These changes were independent of the cytotoxicity of the compounds. Autophagy inhibitor, 3-MA, decreased autophagic protein level in all investigated time periods (Fig 6A and 6B).

### O-DOX and O-DAU increase nSMase activity

**Fig 7:**
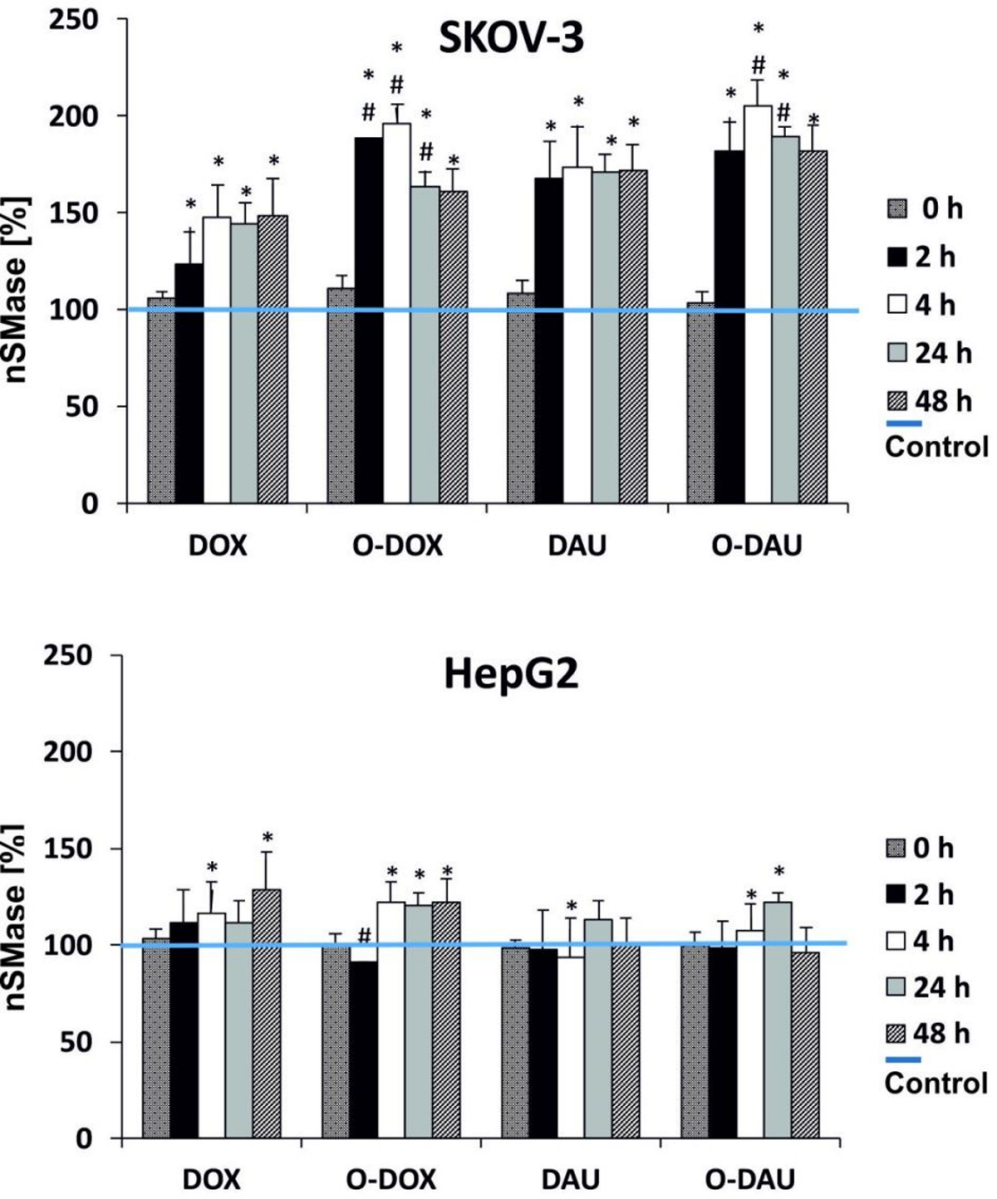
The effect of drugs on nSMase activity in SKOV-3 and HepG2 cells.

In this study we detected changes in nSMase activity as early as after 2 h treatment of cells with test compounds. Treatment of SKOV-3 cells with DOX, DAU and oxazoline derivatives increased nSMase protein levels in a time-dependent manner (Fig 7), with maximal nSMase induction occurring after incubation with O-DOX at 4 h. Higher nSMase levels were produced by O-DOX and O-DAU than the unmodified drugs in all incubation times. Compared to DOX, O-DOX enhanced nSMase levels in SKOV-3 by 65% after 2 h, 48% after 4 h and 20% after 24 h. The greatest differences between DAU and O-DAU were observed after 4 h (30%) and 24 h (20%) of cell treatment.

In liver cancer cells, changes in nSMase activity were detected later (after 4 h incubation) compared to SKOV-3 cells. The derivatives produced some larger changes than the parent compounds. O-DOX increased the level of nSMase to about 120% after 2 and 24 h of treatment, whereas O-DAU stimulated the highest changes of nSMase after 24 h (122%).

### Inhibition of colony formation in response to O-DOX and O-DAU treatment

**Fig 8:**
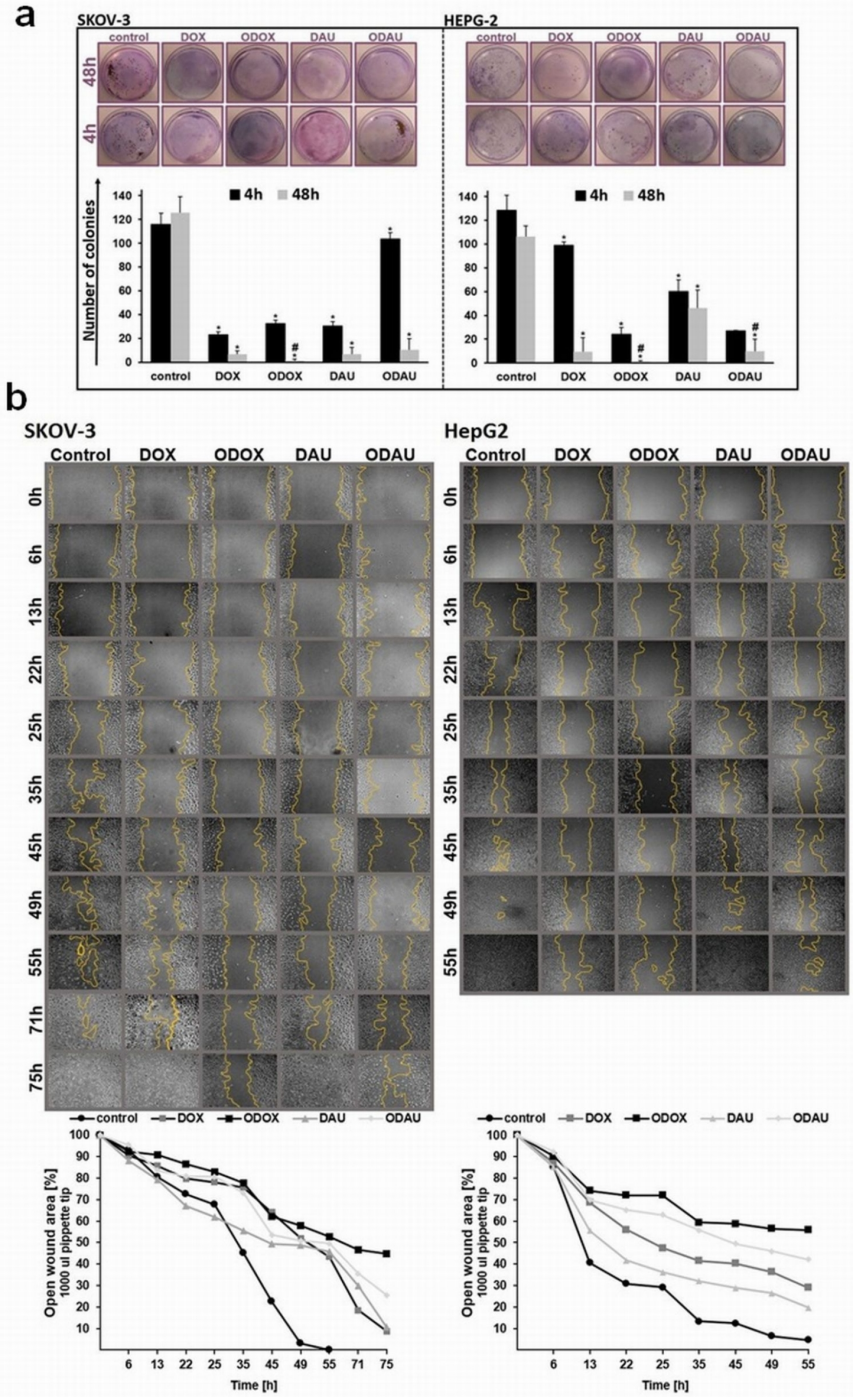
Oxazolines delay the growth of tumor cells and their migration. **(a**) Drugs inhibited colony formation of cancer cells. SKOV-3 (panel left) and HepG2 (panel right) cells were grown in six-well plates (200 cells per well) in triplicates. After 24 h, the culture medium was replaced with fresh medium containing 80 nM of drugs concentration for 4 or 48 h. After treatment each well was washed twice with medium and the experiment continued for 14 days. Surviving colonies were stained (upper panel) and counted (lower panel). (**b**) The effect of drugs treatment on cells migration. Representative photographs of cell scratch test captured from 0 to 75h (SKOV-3) and to 55h (HepG2) after treatment with investigated compounds (Olympus IX70 with phase-contrast, Japan; 100× magnification.) The rate of migration was measured by quantifying the total distance that the cells moved from the edge of the scratch (marked by yellow line) toward the center of the scratch.

Long term in vitro biological characterization was carried out by performing a colony assay, which is an in vitro reliable test for measuring cell survival based on the ability of a single cell to grow into a colony and is frequently used to determine the ability of drugs in killing tumor cells. The results showed in Fig 8A demonstrate that after treatment with tested drugs at a concentration of 80 nM the ability of SKOV-3 and HepG2 cells to form colonies was significantly inhibited. ODOX decreased growth of SKOV-3 cells to approximately 0,85% after 48 h incubation and to 19% after 4h of treatment. In HepG2 cells growth was inhibited to 18% by ODOX and 21% by ODAU after 4h of treatment, whereas extending the incubation time to 48 h lead to cell growth inhibition to 0,9% by ODOX and 9% by ODAU. DOX and DAU showed poorer effect in comparison to oxazoline derivatives. Cell lines were more susceptible to oxazoline derivatives than to parent compounds in the clonal growth assay. A similar tendency was previously observed in the MTT assay.

### Migration of the cells is inhibited by oxazolinoanthracyclines

Cell migration is vital to many physiological and pathological processes including cancer metastasis. For this reason, we used scratch test, where the ability of cells to repopulate a wounded area in a monolayer can be measured. It is worth noting that, (Fig 8B) untreated SKOV-3 cells were highly mobile and repopulated the denuded area within 45 h. However, migration into the wounded area after following O-DOX treatment, migration was greatly abrogated. Ovarian cancer cells did not overgone the wound up to 75 h. Uncovered area after O-DOX treatment was at the level of 44% whereas after DOX and DAU exposition, we observed about 8% and DAU 11% respectively. It should be also taken into account that untreated hepatocellular cancer cells were more mobile than SKOV-3 cells. The fully confluent cells’ monolayer was observed after about 41 hours. Cells treated with O-DOX left uncovered wound area in 56% after 55h of incubation and O-DAU in 42% respectively. Obtained results clearly indicated that, DOX repopulate wound area 27% slower than O-DAU, whereas DAU 22% slower than O-DAU.

## Discussion

Ovarian and liver cancers are solid tumors characterized by high mortality and meagre response to therapy. World Health Organization considered hepatocellular carcinoma (HCC) for the second most common cause of cancer-related deaths worldwide [25]. Ovarian cancer is the most lethal gynecological malignancy among women. Due to the absence of specific symptoms at early stages and lack of early detection, the vast majority of ovarian and liver cancer patients are often diagnosed at an advanced stage. Most of patients have stage III–IV disease at diagnosis [25–27].

Anthracyclines are among the most valuable treatments for various cancers, but their clinical use is limited due to detrimental side effects such as cardiotoxicity, which is clearly related to cumulative dose. The main challenge of pharmaceutical research is to discover less cardiotoxic drugs that maintain their chemotherapeutic effect. Common strategies for preventing cardiotoxic effects include modifying the chemical structures and dosages of anthracyclines [28]. It is known that daunorubicin and doxorubicin generate free radicals both enzymatically, through the mitochondrial respiratory chain, and non-enzymatically through pathways incorporating iron ions [29]. Our research has shown that the oxazoline derivatives generate lower levels of ROS compared to the parent compounds. O-DOX generated a 180% lower level of ROS than DOX after 2 h incubation and O-DAU about 30% less than DAU. In order to evaluate the role of oxidative stress induced by oxazoline derivatives, we estimated the effect of both forms of DOX and DAU on SKOV-3 and HepG2 cancer cells, preincubated with NAC. According to our expectations, preincubation of cancer cells with NAC resulted in a decreased amount of ROS to control levels.

We then estimated the levels of RNS generated by test compounds. Nitric oxide (NO) is a highly reactive molecule within biological systems, capable of interaction with other free radicals, molecular oxygen and heavy metals. NO rapidly reacts intracellularly to form nitrite and nitrate, S-nitroso-thiols or peroxynitrate, and these metabolites are believed to play key roles in mediating many of the NO-associated genotoxic effects [30]. Our results revealed that the oxazoline derivative of DAU in both cancer cell lines induced higher levels of RNS than DAU. There were no differences between DOX and O-DOX. In other data, as a consequence of NO-releasing properties, nitrooxy-doxorubicin has been shown to be less effluxed, accumulate to a higher degree, and have greater cytotoxicity in drug resistant cells [31]. This may be one reason for the increased toxicity induced by modified daunorubicin in HepG2 cells. These observations suggest that generation of oxidative stress is the mechanism of toxicity for both unmodified and modified anthracyclines but it is not the main responsible for the high cytotoxicity of derivatives. We have therefore carried out research on the establishment of a molecular target of derivatives action.

Increases of intracellular ROS and RNS by anthracyclines causes redox imbalance, which ultimately triggers apoptosis [32]. Formation of reactive oxygen species has been shown to modulate nSMase. We have studied whether oxazoline derivatives of anthracyclines are involved in the regulation of nSMase and ceramide generation in ovarian and liver cancer cells. Ceramide, a lipid second messenger, is a prototypic inducer of apoptosis in various cell types [33]. We report that nSMase is upregulated by DOX and DAU. Oxazoline derivatives induced higher changes in nSMase activity in SKOV-3 cells. Our results shed light on the mechanisms of ceramide generation by DOX, DAU and their derivatives.

Another aspect of our current study focused on the biochemical changes occurring during apoptosis induced by oxazoline analogs. ROS signaling and apoptosis are proposed to be mediated by mitochondria [34]. Our data show that a drop in mitochondrial membrane potential occurred, especially at longer incubation times of 24–48 h, following treatment with oxazoline derivatives in cancer cell lines. The changes were larger than those induced by the parent unmodified compounds. Ceramide alters mitochondrial membrane potential, promotes cytochrome-c release and channel formation in the mitochondrial outer membrane. It has been shown that ceramide promotes a rapid fragmentation of the mitochondrial network in a concentration- and time-dependent manner and, as a consequence, the early activation of apoptosis [34].

Increasing interest has recently centered upon programmed cell death other than apoptosis. Importantly, autophagy, which is primarily a protective process, has been linked to both types of cell death, serving either a pro-survival or pro-death function [35]. In fact, autophagy is often referred to as type II programmed cell death (distinct from type I programmed cell death) because it does not require caspase activation or DNA fragmentation, which are classical characteristics of apoptosis [36]. However, it is likely that both processes occur simultaneously, and thus, it is important to understand the signaling pathways that control autophagy, especially when considering that many of the same mechanisms regulate apoptosis. In our previous study, we demonstrated strong cytotoxicity of oxazolinoanthracyclines, particularly O-DOX, in SKOV3 and HepG2 cancer cells [13]. Therefore, in this study we did not only want to identify pathways leading to apoptosis, such as ceramide, but also to understand other routes of cell death induction. To determine whether DOX, DAU or their oxazoline derivatives have an effect on autophagy in SKOV-3 and HepG2 cells, the formation of autophagic vacuoles was detected using fluorescent autophagosome marker. It was demonstrated that all investigated compounds increased the degree of autophagic morphology in cells, but slightly greater changes were observed after treatment with unmodified anthracyclines. In this study, the results clearly indicated that the highest percentage of autophagic cells was found after 48 h treatment with DOX in both investigated cancer cell lines. Consistent with this, fluorescence microscopic analysis showed that typical cellular morphological features of autophagy were present in hepatocellular carcinoma and ovarian carcinoma cells treated with oxazolino-modified and unmodified anthracyclines. Acridine orange (AO) staining was also used, but only as a supplement to show autophagic changes after 48 h treatment with the investigated compounds. It should be kept in mind that acidotropic dyes such as AO are able to detect all acidic vesicular organelles (AVO), which include lysosomes, late autophagosomes and late endosomes. Staining with acridine orange and autophagy assay only indirectly confirm the phenomenon of autophagy. Therefore, we measured LC3 level. The obtained results are consistent with the fluorescence method and confirm a lower level of autophagy in oxazoline-treated cells. Fong et al. found that DOX increased expression levels of LC3 in an epithelial ovarian cancer cell line. In particular, LC3-II is the first mammalian protein identified that specifically associates with autophagosome membranes [37, 38]. The role of autophagy in DOX-induced toxicity is well known. Some studies have used pharmacological agents to manage autophagy [39–41]. On the other hand, literature data have shown that DAU induced a high level of autophagy, which was associated with activation of the extracellular signal regulated kinase ERK1/2. It must be noted that apoptotic cell death induced by DAU was greatly enhanced after inhibition of autophagy by chloroquine and siRNAs targeting Atg5 and Atg7, two of the most important components for the formation of autophagosomes [8, 42].

In our study, we used 3-methyladenine (3-MA), a nucleotide derivative that blocks class III PI3K activity, as an autophagy inhibitor. 3-MA selectively inhibits degradation of endogenous proteins and also acts specifically upon the autophagic/lysosomal degradation pathway [43, 44]. As expected, the results showed that 3-MA decreased autophagic cell death, implying that the analysis was specific and resulted from the action of anthracyclines and oxazolinoanthracyclines.

We found that cellular Ca^2+^ levels rapidly increased upon treatment with modified and unmodified anthracyclines (2 and 4 h), which was accompanied by increased ROS and RNS levels in all cell lines. It is worth noting that ovarian cancer cells and hepatocellular carcinoma cells treated with O-DOX and O-DAU had higher mean levels of intracellular calcium. A growing amount of evidence in recent years shows that ROS and RNS, alongside Ca^2+^, are the main intracellular signal transducers sustaining autophagy [45]. In a specific manner, induction of autophagy requires the production of H_2_O_2_ that oxidizes, among other things, ATG4, an enzyme involved in ATG8 protein maturation and delipidation. This oxidation modification mainly inactivates the delipidation activity of ATG4, leading to an increased formation of LC3-associated autophagosomes [46].

We investigated additionally two major biological activities of tumor cells migration, and colony formation. Metastasis is a process in which primary tumor cells migrate. This process leads to decrease of survival rate of patients. It is important to stop the growth of cancer cells, but also to limit their migration [47, 48]. The DOX and DAU ability to inhibit colony growth and cancer cells migration has been confirmed in numerous studies [49–51]. Our present study shows that in addition to apoptosis induction, O-DOX and O-DAU significantly blocked cell reproductive ability to form a large colony and migration. After 48 h of O-DOX treatment we observed complete suppression of SKOV-3 and HepG2 cells growth, whereas O-DAU was more effective in HepG2 cells.

**Fig 9:**
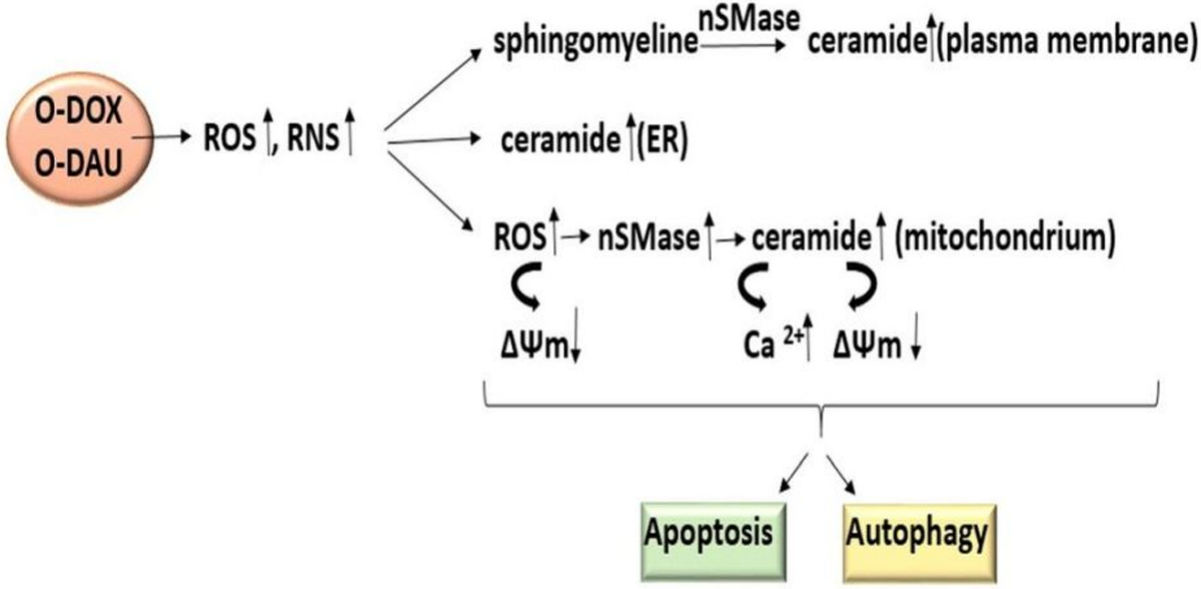
Interference of oxazoline derivatives with signaling pathways in ovarian and liver cancer cells. ER, endoplasmic reticulum; nSMase, neutral sphingomyelinase.

Anthracyclines are still one of the most effective chemotherapeutics due to the wide spectrum of activity. The new derivatives are more cytotoxic despite the induction of a lower level of oxidative stress. It is expected strong anticancer effects from new derivatives and at the same time reduce cardiotoxicity, which has already been demonstrated [3, 52]. We have begun research on the establishment of a molecular target of action of DOX and DAU derivatives which will indicate the mechanisms responsible for strong cytotoxicity (Fig 9). In our study, we used two different cell lines, derived from the group of the most lethal tumors. The ovarian cancer cells were more sensitive to the effects of oxazoline derivatives. Higher levels of intracellular calcium, nitrogen species, nSMase activity, mitochondrial membrane potential depolarization and lower colonies formation were found. The HepG2 cell line proved to be more resistant to the new compounds. HepG2 cells were also more mobile than SKOV-3 cells, which is probably due to the characteristics of this line and its ability to grow as monolayers in small aggregates. Previous studies have also shown that these lines differ significantly in glycoprotein P (P-gp) levels. P-gp is one of the most important multidrug transporters especially in HepG2 cells [53, 54]. Understanding how autophagy and apoptosis pathways maintain the balance between survival and death is crucial to determining the utility of new chemotherapeutics in cancer treatment. Chemical modification at the C3′ position of anthracyclines is a good way to increase the activity against tested cancer cells. This knowledge gives us the opportunity in planning the synthesis of new promising analogs.

Considering all the prior knowledge about oxazolines, it seems that, intensive further investigation of oxazolines analogs could provide a chance to introduce one of them to advanced preclinical trials, and in the future to choose a candidate of powerful anthracycline drug with significantly decrease toxicity.
